# Fully-Connected Neural Networks with Reduced Parameterization for Predicting Histological Types of Lung Cancer from Somatic Mutations

**DOI:** 10.3390/biom10091249

**Published:** 2020-08-28

**Authors:** Kazuma Kobayashi, Amina Bolatkan, Shuichiro Shiina, Ryuji Hamamoto

**Affiliations:** 1 Division of Molecular Modification and Cancer Biology, National Cancer Center Research Institute, 5-1-1 Tsukiji, Chuo-ku, Tokyo 104-0045, Japan; abolatka@ncc.go.jp (A.B.); rhamamot@ncc.go.jp (R.H.); 2 Cancer Translational Research Team, RIKEN Center for Advanced Intelligence Project, 1-4-1 Nihonbashi, Chuo-ku, Tokyo 103-0027, Japan; 3 Department of NCC Cancer Science, Graduate School of Medical and Dental Sciences, Tokyo Medical and Dental University, 1-5-45 Yushima, Bunkyo-ku, Tokyo 113-8510, Japan; 4 Department of Diagnostic Imaging and Interventional Oncology, Graduate School of Medicine, Juntendo University, 2-1-1 Hongo, Bunkyo-ku, Tokyo 113-8421, Japan; ivo@juntendo.ac.jp

**Keywords:** deep learning, Diet Networks, lung cancer, interpretable neural networks

## Abstract

Several challenges appear in the application of deep learning to genomic data. First, the dimensionality of input can be orders of magnitude greater than the number of samples, forcing the model to be prone to overfitting the training dataset. Second, each input variable’s contribution to the prediction is usually difficult to interpret, owing to multiple nonlinear operations. Third, genetic data features sometimes have no innate structure. To alleviate these problems, we propose a modification to Diet Networks by adding element-wise input scaling. The original Diet Networks concept can considerably reduce the number of parameters of the fully-connected layers by taking the transposed data matrix as an input to its auxiliary network. The efficacy of the proposed architecture was evaluated on a binary classification task for lung cancer histology, that is, adenocarcinoma or squamous cell carcinoma, from a somatic mutation profile. The dataset consisted of 950 cases, and 5-fold cross-validation was performed for evaluating the model performance. The model achieved a prediction accuracy of around 80% and showed that our modification markedly stabilized the learning process. Also, latent representations acquired inside the model allowed us to interpret the relationship between somatic mutation sites for the prediction.

## 1. Introduction

With the advance of big data in biomedicine, deep learning has achieved state-of-the-art performance in various fields, including bioinformatics. A large number of analytic pipelines—such as sequence analysis, protein structure estimation, molecular property or interaction prediction, and biomedical image analysis—have incorporated deep-learning-based algorithms [1]. One remarkable feature of deep learning is that it excels at handling raw data in an end-to-end manner, acquiring the essential high-level features automatically [2]. Thus, the model can learn features meaningful for distinguishing attributes of samples without relying on feature engineering based on domain knowledge. As human experts do not always know which feature representation best suits a given task, deep learning can shed light on machine learning tasks, particularly those involving complex biological phenomena. Moreover, its scalability enables it to handle the processing of massive quantities of data [3].

Cancer is a leading cause of death worldwide. Complex intra- and inter-layer interactions between omics, such as somatic mutation, gene expression, copy number alteration, and deoxyribonucleic acid (DNA) methylation, influence its biological behavior. One of the main purposes of cancer genome analysis is to clarify the relationship between genetic variations and phenotypes underlying cancer’s biology. Currently, genome-wide association studies (GWAS) are the most widely used technique for analyzing genotype–phenotype associations based on a statistical test to determine the level of association between a single genetic variant and a phenotype. However, suppose there are epistatic interactions between genetic variants associated with phenotypes. In that case, the association cannot be identified through GWAS because it tests each gene locus independently for association with a phenotype of interest [4]. Such complex compositions also hinder the power of conventional machine learning techniques by making it difficult to design custom features, which are prerequisites for most of the algorithms. Therefore, deep learning should be a promising approach to successfully handling the mapping between genotype and phenotype, leading to the data-driven discovery of the critical signatures of somatic mutations involved in cancer genesis.

### 1.1. Current Challenges in Applying Deep Learning to Genomic Data

To the best of our knowledge, there have been only a few studies that utilize deep learning to analyze the genotype–phenotype association. This is because some fundamental challenges arise when deep neural networks are applied to identify genetic characteristics associated with cancer phenotypes.

The first obstacle is the substantial imbalance between the number of samples and the number of features per sample. In other words, the number of genetic features or covariates, which typically ranges in the millions, sometimes exceeds the number of patients. Under such circumstances, deep neural networks tend to overfit the training data, failing to generalize well about unseen data. This is because deep neural networks are usually in an over-parameterized condition, in which a vast number of free parameters must be optimized by backpropagation. One straightforward solution is to design a lightweight architecture that employs fewer parameters without sacrificing representational capacity. Another approach is to use regularization methods—including dropout, early stopping, and weight decay—that imposes some penalty on the model to reduce its test error but not its training error [5]. Notably, multi-task learning is a special type of regularization [6]. Some researchers integrate auxiliary tasks, which can leverage additional information, including domain knowledge or self-supervision based on unlabeled data, as implicit regularization methods [7,8,9].

Second, deep neural networks are most often treated as a black-box function, and it is difficult to provide a human-understandable interpretation of its prediction. In particular, many researchers in the field of biomedicine are interested more in the biological insights, such as genetic variants associated with a cancer phenotype, than in the model accuracy. Therefore, difficulty in the interpretability of a deep learning model can be a major drawback. A straightforward approach for interpreting a model’s behavior is to systematically vary each feature of the input and observe how the output changes. A more computationally tractable method utilizes the derivative or gradient, observing the sensitivity to small perturbations as an indicator of the importance of the input feature [10,11]. Other algorithms, such as Local Interpretable Model-agnostic Explanations, create a linear approximation of any classifier or regressor for a local neighborhood of given input [12]. Moreover, embedding techniques can provide insights into how the model captures each input feature in a particular context by distributed representation, reflecting the semantic relationship between variables [13,14].

The last problem to be addressed here is the availability of innate structure in the genetic data features. When DNA base sequences are given as input, deep learning architectures such as convolutional neural networks (CNNs) and recurrent neural networks (RNNs) can predict some functional activities based on the sequential information [15,16,17,18,19]. However, there are some types of biological data for which no spatial or sequential structure can be objectively defined. For example, when data for a somatic mutation such as single-nucleotide variants (SNVs) are acquired through genotyping techniques, it is difficult to assign any meaningful elemental arrangement to the array representing the correspondence between genomic position and the presence of mutations. One unique approach for alleviating this problem is transforming non-image genomic data into image form, thereby integrating the advantage of CNNs [20]. However, the arbitrary transforming of genomic data into images can itself be regarded as a feature engineering, lacking a strong rationale for its optimal formulation. Therefore, the naive implementation of a multilayer perceptron (MLP) consisting of fully-connected layers has been used as the first choice. Still, this approach is problematic because the number of free parameters in the first layer, which is the product of the number of input features and the number of hidden units, can be quite large.

### 1.2. Related Work

To conduct genotype–phenotype association studies using a deep-learning-based approach, dimensionality reduction (including auto-encoders) or preselection is generally applied to reduce the number of effective input variables. However, dimensionality reduction or preselection can overlook variables that have a small effect. Also, the impact of the individual input variable on the prediction gets more challenging to measure due to the abstraction through these pre-processing techniques. Recently, Romero et al. proposed the *Diet Networks* architecture to reduce the number of free parameters to be learned [21]. By exploiting the transposed data matrix (which is similar to considering features as samples and vice versa) for auxiliary networks, it approximates a part of model parameters without keeping gradient information, thus mitigating computational loads. Diet Networks consist of layers that are fully-connected, which enables to handle raw genomic data without any assumption regarding their innate structure. Nevertheless, despite the well-formulated learning framework of Diet Networks, it might lose the capacity to learn the meaningful relationship between input features and given labels, particularly when the pattern of variables in the transposed data matrix is limited.

### 1.3. Our Contributions

Given the challenges above of deep learning in the field of bioinformatics, we modified the original Diet Networks concept by adding *element-wise input scaling (EIS)*. The core of our modification is to relax the formulation of Diet Networks by introducing a small number of learnable parameters that can be optimized by usual backpropagation. Hence, the dependency on the transposed data matrix should be mitigated to improve the learning capacity of the model. To investigate the practical performance of the proposed method compared with other configurations of Diet Networks and MLP with the same architecture, we defined a simple task—predicting the histological types of lung cancer from somatic mutations (i.e., SNVs, insertions and deletions). Notably, the introduction of EIS led to an apparent effect that helped stabilize the model in the training process under our experimental setting. Based on the best configuration, the prediction accuracy of Diet Networks with EIS reached at around 80%, which was the same level as the MLP. Our formulation of the task also allowed us to observe each input variable’s internal representation from the trained model parameterization. Interestingly, the internal representations were highly compressed into a narrow manifold. Then, the prediction capacity of the model was approximated by the two-dimensional (2D) subspace spanned by the first and second principal components (PCs). Finally, we confirmed that PC scores, according to a particular axis, can be interpreted as an indicator of each somatic mutation site’s relevance to the histological types.

## 2. Materials and Methods

### 2.1. Data Collection

Information regarding lung cancer histology and somatic mutations was downloaded from the Pan-Lung Cancer dataset [22], which is publicly available at cBioPortal (http://cbioportal.org). From among the 1114 patients in the dataset, we selected 954 patients with clinical information, including 481 with lung adenocarcinoma (LUAD) and 473 with lung squamous cell carcinoma (LUSC). To obtain the equal size of five splits, the last four cases were excluded from the dataset, resulting in final population size of 950 patients. As a result, each split has the same number of patient (n = 190) for the subsequent 5-fold cross-validation. Two histological types (LUAD and LUSC) were used as a binary class label for the prediction task. As all the data analyzed in the present study are in the public domain, ethical approval was not required.

### 2.2. Preprocessing of Somatic Mutation Data

A total of 17,961 unique gene symbols were identified from the dataset. The preprocessing pipeline was applied as follows. First, the number of somatic mutations—such as SNVs, insertions, and deletions–was counted for each gene symbol. Note that we did not consider genome structural variants such as copy number alterations and fusing genes. We also did not count somatic alterations that occurred at silent or spliced regions of the genome, as it can exert minimum biological impact. Then, the genes were arranged in order by mutation count in the concatenated data matrix along with the samples. Lastly, values associated with the genes were binarized according to the presence of any somatic mutation. If a gene had a positive mutation count, a value of 1 was assigned, and 0 was assigned otherwise.

### 2.3. Proposed Methods

In this study, we aimed to identify improved configurations and modifications for the original Diet Networks concept [21] from the viewpoint of the model’s learning ability. Here, we first review the original Diet Networks concept and then describe our contributions. The source code and the data employed in this work are publicly available on GitHub (https://github.com/Kaz-K/diet-networks).

#### 2.3.1. Overview of Diet Networks

**Figure 1:**
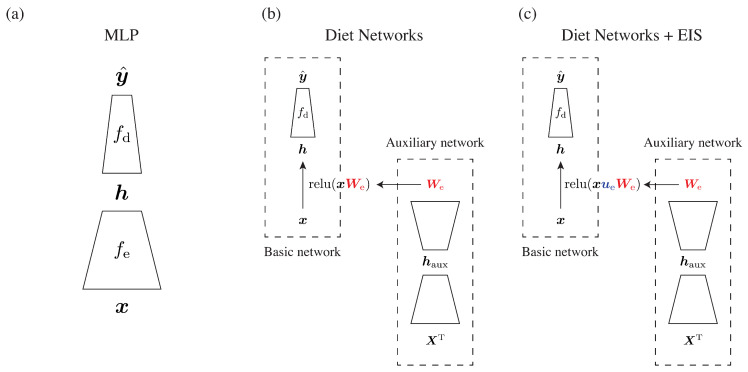
Overview of network architectures investigated in the present study. (**a**) Multilayer perceptron (MLP) consists of three-layered fully-connected networks; its overall architecture is the same as that of the basic network of Diet Networks. (**b**) The original Diet Networks concept approximates two fat layers, $W_{e}$ and $W_{r}$ (only $W_{e}$ is shown here), by the auxiliary networks taking the transposed data matrix as their input. (**c**) Our modification to Diet Networks. Applying element-wise input scaling (EIS) provides a learnable vector $u_{e}$ to the input, which is directly optimized through backpropagation of gradients from the output $\hat{y}$ to the input x. On the other hand, $W_{e}$ is estimated by the auxiliary network.

Suppose that there is a substantial imbalance between the number of samples *N* and the number of features $N_{d}$ ($N\ll N_{d}$). The Diet Networks concept aims to reduce the number of parameters in a fully-connected neural network given a data matrix $X\in R^{N\times N_{d}}$ with *N* samples and $N_{d}$ features. It consists of three components: one basic network *F* and two auxiliary networks $G_{e},G_{r}$ (Figure 1). Each component is built on fully-connected layers, a structure that can be versatile and effective for uncovering complex genotype–phenotype patterns [23]. For simplicity, we consider a particular case in which all the networks are three-layered MLPs. Given an input matrix X, the basic network computes corresponding hidden layer $H\in R^{N\times N_{h}}$ via an encoding part of the network $f_{e}$, and then outputs $N_{c}$-class classification $\hat{Y}\in R^{N\times N_{c}}$ through a discriminative part $f_{d}$. Note that each network consists of a linear transformation and a nonlinear activation function. Optionally, the basic network has a reconstruction path $f_{r}$ to reconstruct the input $\hat{X}\in R^{N\times N_{d}}$, which is bifurcated from the hidden layer. Thus, the formulation of the basic network can be described as follows:

(1) $$\hat{Y}=f_{d}\left(H\right),\;\hat{X}=f_{r}\left(H\right),\;H=f_{e}\left(X\right).$$

Let $W_{e}$ and $W_{r}$ be affine transformations of $f_{e}$ and $f_{r}$, respectively. Given the data matrix $X\in R^{N\times N_{d}}$ and corresponding hidden layer $H\in R^{N\times N_{h}}$, the size of $W_{e}$ and $W_{r}^{T}$ will be $N_{d}\times N_{h}$. Therefore, the dimensionality of these matrices can grow linearly with that of the input data, a phenomenon known as a *parameter explosion* [21]; this causes difficulties in scaling neural networks for handling samples with a very large number of attributes. Based on these observations, Romero et al. refer to $W_{e}$ and $W_{r}$ as a *fat hidden layer* and a *fat reconstruction layer*, respectively [21].

Two auxiliary networks are introduced to alleviate parameter explosions in the basic network. These auxiliary networks take a transposed data matrix $X^{T}\in R^{N_{d}\times N}$ as input. Then, the corresponding fat parameters are calculated by the auxiliary networks as follows:

(2) $$W_{e}=G_{e}\left(X^{T}\right),\;W_{r}=G_{r}\left(X^{T}\right).$$

Note that now the weights of fat layers are obtained as the outputs of these auxiliary networks. Then, computational loads can be drastically reduced because gradient information for each matrix weight does not need to be kept during the model training. Also, this estimation is based on the assumption that each variable’s feature may be associated with the pattern of values taken across the patients. Therefore, it doesn’t need to be a transposed counterpart to the basic network. In other words, any pattern of values from the same data distribution can also be useful.

Finally, the overall model is trained by minimizing the following objective function:

(3) $$L=H\left(\hat{Y},Y\right)+\gamma ||\hat{X}-X{\left|\right|}_{2}^{2},$$

where H indicates a cross-entropy function and γ is a hyperparameter to balance classification loss and reconstruction loss.

#### 2.3.2. Element-Wise Input Scaling for Neural Networks

The original Diet Networks concept is well-formulated for handling the parameter explosion problem; however, the learning capacity could be heavily dependent on the transposed data matrix. There is no room to directly optimize the weights of the first affine layer by backpropagation, which is a standard learning algorithm for deep learning. Therefore, if the pattern of values inside the transposed data matrix does not have sufficient variation for a given task, the representation ability of the model might be rigorous, failing to capture the data’s characteristics enough for the prediction. Thus, we aimed to relax the formulation by assigning an additional degree of freedom to the networks without significantly increasing the parameters.

We assigned learnable variable-wise scalars as EIS by imposing a diagonal metric on the input space, which is represented as a diagonal matrix, $U_{e}\in R^{N_{d}\times N_{d}}$. If sparsity is encouraged using a proper norm (e.g., L1) on the scale factors, feature selection can also be achieved in the formulation. Similarly, we also added a diagonal matrix $U_{r}\in R^{N_{d}\times N_{d}}$, which is expected to compensate for the representative capacity of $W_{r}$. As a whole, the formulation of *Diet Networks with EIS* can be presented as follows:

(4) $$\hat{Y}=f_{d}\left(H\right),\;\hat{X}=\mathrm{sigmoid}\left(HW_{r}U_{r}\right),\;H=\mathrm{relu}\left(XU_{e}W_{e}\right).$$

Note that the optimization processes for the diagonal matrices, $U_{e}$ and $U_{r}$, and affine matrices, $W_{e}$ and $W_{r}$, are quite different. As shown in Figure 1, the former diagonal matrices are optimized by backpropagation through the gradient from the output $\hat{Y}$ to the input $\hat{X}$, which is in the usual manner of deep learning models. The latter affine matrices are estimated by the auxiliary networks in the same way as the original implementation without holding gradient information to alleviate the computational burden. Since the diagonal matrix has only $N_{d}$ effective parameters to be learned, we consider that the total computational load does not increase significantly.

We also extended the objective function by adding an L1 penalty to the scale factors as follows:

(5) $$L=H\left(\hat{Y},Y\right)+\gamma ||\hat{X}-{X||}_{2}^{2}+\delta ||U_{e}{\left|\right|}_{1},$$

where γ and δ are weights for balancing the importance of the terms.

In addition to its use with Diet Networks, EIS can be applied to MLP as well. Hereinafter, this modified MLP architecture is referred to as *MLP with EIS*.

#### 2.3.3. Implementations of Neural Networks

For comparison, we implemented four types of fully-connected neural networks: MLP, MLP with EIS, Diet Networks, and Diet Networks with EIS. As the MLP architecture is the same as that of the basic network of Diet Networks, we will use the same notation for describing the structure of the MLP. All networks shared a basic network consisting of an input layer of $N_{d}$ nodes, a hidden layer of $N_{h}$ nodes, and $N_{c}$ output nodes for the given classification task. The two auxiliary networks of Diet Networks were designed with an input layer of *N* nodes, a hidden layer of $N_{j}$ nodes, and an output layer of $N_{h}$ nodes.

### 2.4. Analysis of Hidden Representations

Let $x_{i}$ be one data sample, which is given as the *i*th row of the data matrix X. The corresponding hidden representation $h_{i}$ can be regarded as the *i*th column of $W_{e}$ in the MLP and Diet Networks. From the same point of view, the *i*th column of $U_{e}W_{e}$ can be taken as the *i*th hidden representation $h_{i}$ in Diet Networks with EIS. This simplification can be made because the data matrix is binarized, containing only 0 s and 1 s (see Section 2.2). We visualized the distribution of hidden representations of somatic mutations acquired in each neural network to observe how each somatic mutation was embedded and contributed to the overall output. A t-distributed stochastic neighbor embedding (t-SNE) projection [24,25] and PCA plotting were performed for the 2D visualization.

### 2.5. Performance Evaluation

The study dataset (n=950) was split into five groups. For each split of 190 cases, one group was taken as a validation dataset (n=190), and the remaining four groups were used as a training dataset (n=760) for the model. In each epoch of training, the accuracy of the histology’s binary classification was evaluated using the validation dataset, and the accuracy score was retained. After repeating this procedure five times (5-fold cross-validation), the accuracy for a particular period was gathered for all procedures, and the mean and standard deviation were computed for each model configuration.

## 3. Results

### 3.1. Experimental Setup

All neural networks (i.e., MLP, MLP with EIS, Diet Networks, and Diet Networks with EIS) were implemented using Python 3.7 with PyTorch library 1.2.0 [26] on an NVIDIA Tesla V100 graphics processing unit with CUDA 10.0. According to the dataset and the binary classification task, basic conditions were as follows: N=950, $N_{d}=17,761$, and $N_{c}=2$. Adam optimization [27] was used with initial learning rates of $5\times 10^{-3}$. A weight decay of $5\times 10^{-4}$ was applied. The other hyperparameters were empirically determined as follows: $N_{h}=128$, and $N_{j}=256$; the batch size was 100, and the maximum number of epochs was 5000. Note that the transposed matrix $X^{T}\in R^{17,761\times 950}$ was fixed during training, whereas an input matrix for each iteration was split according to the batch size to be a size of 100×17,761. The magnitudes of γ and δ were tuned in the patterns of {0.001,0.01,0.1} and {0.0001,0.001,0.01}, respectively. In the condition without the reconstruction path, the numbers of parameters of each network architecture (i.e., parameters of $f_{e}$ and $f_{d}$ in MLPs, and those of $f_{e}$, $f_{d}$, and $G_{e}$ for Diet Networks) are shown in Table 1. Note that the increase in the number of parameters of Diet Networks with EIS (Baseline) is small compared to that of Diet Networks (Baseline).

### 3.2. Evaluation of Classification Accuracy

The classification accuracy of MLP, MLP with EIS, Diet Networks, and Diet Networks with EIS using various hyperparameter settings is presented in Table 2. Mean accuracy with standard deviation was calculated during the period between epochs 400 and 500. “Baseline” indicates that both γ and δ were set to 0. The weight for the reconstruction error γ was varied, and the value of 0.1 exhibited the best accuracy (0.78±0.05) for Diet Networks (marked in the table with an asterisk). Similarly, a γ value of 0.1 provided the highest accuracy (0.79±0.02) for Diet Networks with EIS (marked with a double asterisk). Positive values of δ to encourage the sparsity of EIS did not improve the classification accuracy. The best configurations for both MLP (marked with a dagger) and MLP with EIS (marked with a double dagger) are also indicated in Table 2.

### 3.3. Observation of Learning Process

**Figure 2:**
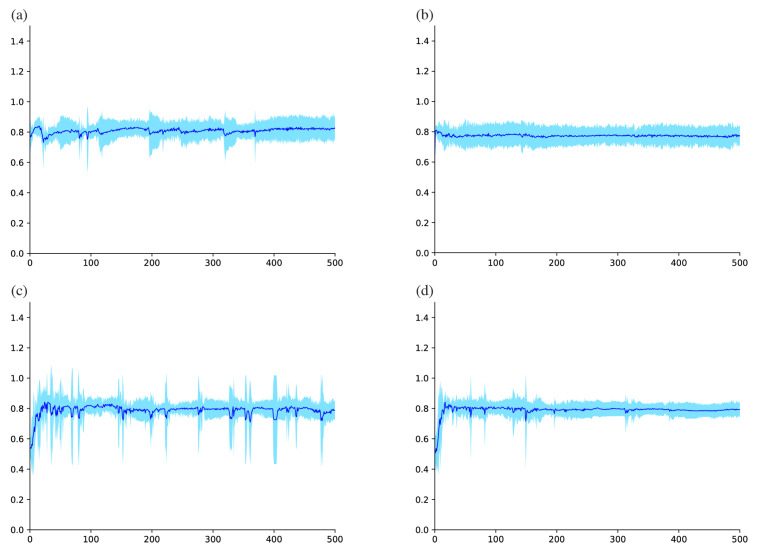
Training curve showing validation accuracy for each model during a short period between epochs 1 and 500: (**a**) multilayer perceptron (MLP) (baseline), (**b**) MLP with element-wise input scaling (EIS) (baseline), (**c**) Diet Networks (γ=0.1), and (**d**) Diet Networks with EIS (γ=0.1). Note that a relatively broad range of variance appeared in the training curve of Diet Networks (γ=0.1). Vertical axis and horizontal axis indicate accuracy mean ± standard deviation and number of epochs, respectively.

**Figure 3:**
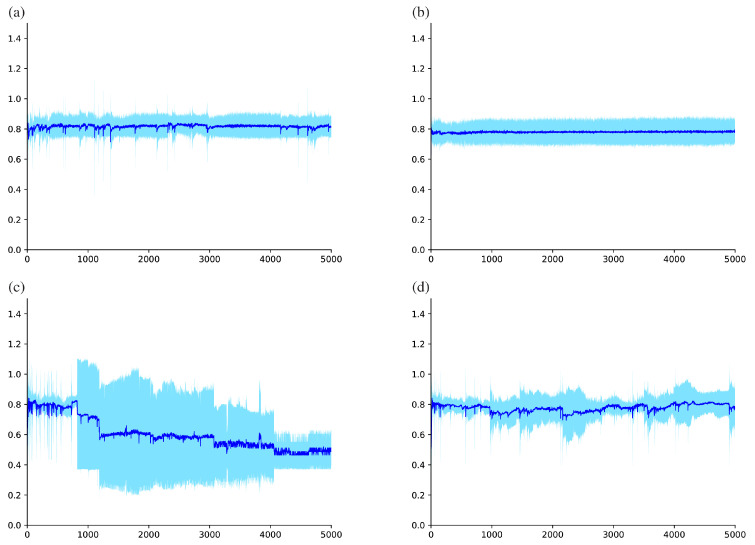
Training curve showing validation accuracy for each model during a long period between epochs 1 and 5000: (**a**) multilayer perceptron (MLP) (baseline), (**b**) MLP with element-wise input scaling (EIS) (baseline), (**c**) Diet Networks (γ=0.1), and (**d**) Diet Networks with EIS (γ=0.1). Note that the classification accuracy of Diet Networks gradually dropped to around 0.5 as training proceeded. Vertical axis and horizontal axis indicate accuracy mean ± standard deviation and number of epochs, respectively.

The training curves of each model architecture under the best configuration were evaluated. For these models, Figure 2 displays the mean with the standard deviation of validation accuracy during the shorter training process between epochs 1 and 500, while Figure 3 displays the more extended period between epochs 1 and 5000. These observations show that MLP (Baseline), MLP with EIS (Baseline), and Diet Networks with EIS (γ=0.1) achieved relatively stable training processes. On the other hand, the training curve of Diet Networks (γ=0.1) was unstable right after the start of the training, which can be seen as relatively large variances in Figure 2c. Especially after around epoch 1000, the prediction performance markedly degraded and eventually dropped to near the chance rate (Figure 3c). The same unstable training trend was reproduced for all configurations of Diet Networks without EIS, and the addition of EIS could improve the stability of the learning process for each (see the difference in standard deviations between model configurations in Table 2).

### 3.4. Distribution of Hidden Representations

**Figure 4:**
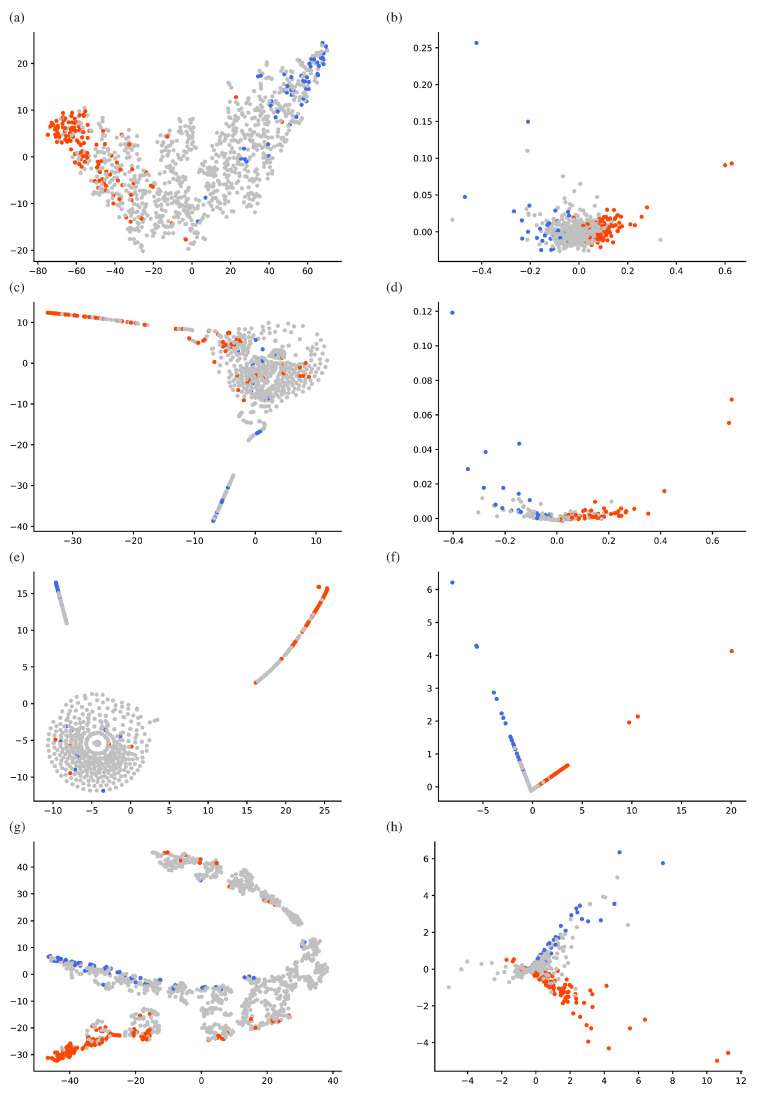
Distribution of hidden representations: (**a**) t-distributed stochastic neighbor embedding (t-SNE) projection for multilayer perceptron (MLP) (baseline), (**b**) principal component analysis (PCA) plot for MLP (baseline), (**c**) t-SNE projection for MLP with element-wise input scaling (EIS) (baseline), (**d**) PCA plot for MLP with EIS (baseline), (**e**) t-SNE projection for Diet Networks (γ=0.1), (**f**) PCA plot for Diet Networks (γ=0.1), (**g**) t-SNE projection for Diet Networks with EIS (γ=0.1), and (**h**) PCA plot for Diet Networks with EIS (γ=0.1). Horizontal axis and vertical axis of PCA plots indicate the first and second principal components, respectively. Lung adenocarcinoma (LUAD)-dominant genes and lung squamous cell carcinoma (LUSC)-dominant genes are colored red and blue, respectively.

The hidden representations of these four types of model architecture based on the obtained configurations were evaluated by t-SNE projection and PCA plotting in 2D space. For each architecture, trained models in the first split in the 5-fold cross-validation procedure were evaluated at epoch 500. Further, to interpret the plots, we evaluated statistically significant (*p* < 0.05) frequent somatic mutations for each histology by using the t-test, and classified each gene into two groups (Table 3): *LUAD-dominant* and *LUSC-dominant*. For example, the LUAD-dominant group includes genes with somatic mutations that occurred statistically frequently in adenocarcinoma histology. In this manner, a total of 482 genes were classified as LUSC-dominant, and 540 as LUAD-dominant. In 2D plots of the hidden representations (Figure 4), each mutation site is colored according to these groups. Notably, the directional preference of a cluster can be understood as the preference of each gene for each histology.

### 3.5. PCA Approximation

As can be seen in the PCA plots in Figure 4, there is a directional preference of hidden representations, which implies that embedded variables are aligned on a narrow manifold. We approximated each hidden representation $h_{i}$ based on the linear combination of PCs as follows:

(6) $$h_{i}\approx \sum_{k\in K}s_{k}\times {\mathrm{PC}}_{k},$$

where $s_{k}$ is the k-th PC score of $h_{i}$ for the k-th principal component ${\mathrm{PC}}_{k}$ and K is a set of indices of PCs. Here, we compared three patterns of indices: K∈{(1),(2),(1,2)}.

**Figure 5:**
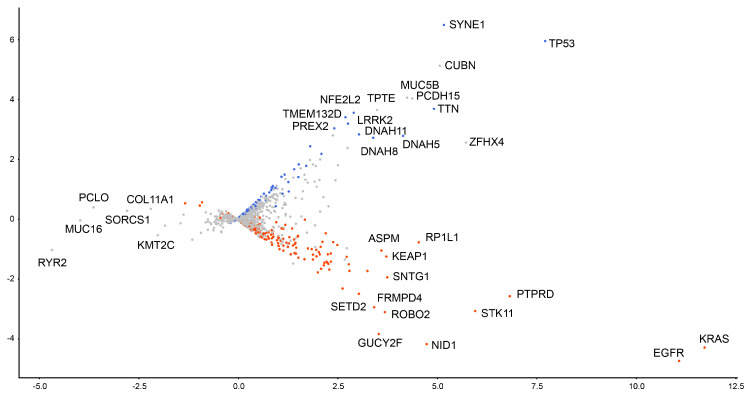
Principal component analysis (PCA) plots of hidden representations with names of genes for Diet Networks with element-wise input scaling (EIS) (γ=0.1). Horizontal axis and vertical axis indicate the first and second principal components, respectively. Lung adenocarcinoma (LUAD)-dominant genes and lung squamous cell carcinoma (LUSC)-dominant genes are colored red and blue, respectively.

Using this approximation, we evaluated validation accuracy by applying the same 5-fold cross-validation (Table 4). Suppose an approximated model can reproduce the same level of prediction accuracy with the non-approximated one. In that case, we can consider that the relationship between hidden representations in the decomposed subspace is representative enough for the model output. Interestingly, the 2D PCA approximation (K=(1,2)) of Diet Networks with EIS produced an accuracy of 0.80±0.02, which was very similar to the non-approximated result of 0.79±0.02 (see the row indicated by the double asterisk in Table 2). The same tendency was also confirmed in MLP (Baseline) and MLP with EIS (Baseline), while only the approximated Diet Networks (γ=0.1) was unable to achieve the original level of prediction accuracy, showing a performance drop from 0.78±0.05 to 0.67±0.11. Therefore, we can consider that the 2D PCA plot of the approximated Diet Networks with EIS (γ=0.1) represented a significant relationship between variables for the model output (Figure 4h). Note that the 2D relationship between gene mutations can be easily understood using visualization. We also show the same PCA plots with some gene symbols in (Figure 5).

Moreover, we investigated the dominant PC for the model output by comparing the classification accuracy between K=(1) and K=(2) based on Diet Networks with EIS (γ=0.1). In this particular case, the second PC can be more representative for the preference of each gene because the approximation by K=(2) produced a higher accuracy (0.66±0.13) than that of K=(1) (0.56±0.12). This demonstrates that each feature’s importance can be estimated by the corresponding PC score for ${\mathrm{PC}}_{2}$. Therefore, the positive and negative directions of ${\mathrm{PC}}_{2}$ can be regarded as preferences for LUSC and LUAD, respectively. The PC scores for somatic mutations with large positive or negative values are listed in Table 5.

Note the similarities and differences between the lists of genes in Table 3 and Table 5. While the two lists were not entirely consistent, there was a considerable overlap between them. For example, LUSC-dominant genes, such as *TP53*, *TTN*, *NFE2L2*, and *LRRK2*, and LUAD-dominant genes, such as *KRAS*, *STK11*, *EGFR*, *PTPRD*, *NID1*, and *FRMPD4*, were also shown in the list of positive and negative PC scores, respectively, in Table 5. Other genes were not shared between these lists, implying that each measure was weighted differently for individual genes.

## 4. Discussion

One remarkable consequence obtained by adding EIS was the stabilized training process of Diet Networks (Figure 3), which maintained the same level of classification accuracy as MLPs with the same architecture (Table 2). The stability of the training process of the deep learning model is quite important. If the learning curve shows oscillation, it means that the model is not converged to the optimal solution. This benefit may owe to the fact that EIS provides the network with an additional degree of freedom, particularly for Diet Networks. Because the original Diet Networks concept does not allow direct propagation of gradients to fat layers, the representation capacity tightly depends on the fixed pattern of values in the dataset as given by the transposed data matrix. Therefore, when the variation of values in the transposed data matrix is not enough, it can be challenging to capture meaningful hidden representations for a particular task, impairing the learning capacity of the model, as shown in (Figure 3c). Because the number of parameters of Diet Networks with EIS is still much smaller than that of MLPs (Table 1), this empirical technique to add EIS can be useful in expanding the application of Diet Networks to other machine learning tasks. This perspective may be particularly important in the field of biomedicine since a discordance between the number of samples and the high dimensionality of features per sample is common when handling genetic data.

We also investigated the interpretability of the models by using PCA approximations. Interpretability is the ability to provide the meaning in a manner understandable to a human [28]. Providing an interpretable view into how the model works can be more important than a simple binary prediction. Identifying specific factors that influence the phenomenon can contribute to new treatments and more precise diagnoses in the field of biomedicine. In our experiment, the 2D PCA approximation reproduces the predictive performance of Diet Networks with EIS at a rate of nearly 100% (Table 4). The overall performance was also sufficiently high (0.80±0.02) under the approximation. This ensured that 2D subspaces spanned by the first and second PCs are representative of the classification model and do not oversimplify the essential features. This decomposability mapped every somatic mutation site to be readily interpretable in the hidden space, where the positional relationship between genes indicates how the model treats each gene in relative terms for the classification (Figure 5).

Furthermore, we pursued the interpretation for the directional preference in the subspace. In our findings, the higher reproducibility rate of the 1D PCA approximation along the second PC direction suggested that the PC scores for ${\mathrm{PC}}_{2}$ can estimate the importance of each factor for the model output (Table 5). Interestingly, there was a considerable overlap with gene lists according to the frequency information (Table 3). For genes that were shared between the lists according to frequency measure and PC score, we can speculate that their frequency information has a significant impact on the model prediction of Diet Networks with EIS. Still, there is also a discrepancy between two lists, and only one PC direction was unable to provide a sufficient approximation for the classification accuracy. Therefore, we also noticed that Diet Networks with EIS can take into account not only frequency information but also the effects of interactions between features for particular genes. Intuitively, the interaction between variables is apparent because a lot of genes distributed out of perpendicular to the PC axes (Figure 5).

An interesting question is whether the somatic mutation sites with higher PC scores indeed have biological meanings, especially those already known to exert biological functions in lung cancer, according to other references in the literature. For example, among somatic mutation sites showing top 10 negative PC scores (Table 5b), *KRAS*, *EGFR*, *STK11*, and *SETD2* have already been shown to be significantly mutated genes for LUAD, and, more importantly, the majority of these genes seem to be mutated exclusively in LUAD and not in LUSC [22]. Similarly, another study showed that *STK11* and *KRAS* mutations—all holding negative PC scores—are associated with much higher frequencies in LUAD than in LUSC. Notably, *KRAS* has shown mutation with a frequency 26 times higher in LUAD than in LUSC [29]. As for the preference of LUSC (Table 5a), *NFE2L2*, which has the eighth largest positive PC score, has been reported as a significantly mutated gene in LUSC [22]. Generally, few known somatic mutations occur exclusively in LUSC and not in LUAD, and overlapping mutations sometimes occur among different histology types [22]. What needs to be discussed carefully here is that *TP53*, *ZFHX4*, and *MUC5B* are known to be significantly mutated in both LUAD and LUSC [22,30]. For example, mutations in *MUC5B* have also been detected in both LUAD and LUSC [31]. As far as we observed, the majority of these genes mutated in both subtypes belong to the positive PC score group (see Figure 5 for *ZFHX4*). We thought that the two-sided preference of mutation sites would be reflected by relatively large norms to the direction of the first PC; however, it was not exclusive for the particular set of genes because *KRAS* and *EGFR* also showed relatively high first PC scores, for example. Therefore, at least for the genes responsible for LUAD, we can conclude that the decomposed hidden representations reflect the histological preference, which is particularly shown by the second PC scores. From a more fundamental point of view, interpreting the hidden representations inside each neural network depends on the level of genetic information available, regularization techniques, and learning tasks. We expect that if the analysis is further integrated with other bioinformatics pipelines, this method may provide a data-driven approach to finding cancer-type-specific driver candidates.

The task of predicting the histological type of lung cancer from somatic mutations (i.e., SNVs, insertions, and deletions) is simple yet fundamental to the practice of cancer medicine. Compared with other biological data such as those for gene expression, somatic mutations are particularly useful in classifying tumors because they are more robust to variations in environmental or experimental conditions. Besides, if some combinatorial somatic mutation patterns could be identified that can predict cancer types or subtypes, it would be essential to develop diagnostic gene marker panels, facilitating personalized medicine. On the binary classification task of identifying whether the somatic mutation data are associated with squamous cell carcinoma or adenocarcinoma, the proposed model achieved a prediction accuracy of around 80%. Deep learning models that provide both high accuracy and interpretability may also be useful in precision medicine.

### 4.1. Limitations

Our experiments on Diet Networks with EIS have the following limitations. First, we did not evaluate whether the same interpretable hidden interpretations can be obtained from different datasets. The task of binary classification from somatic mutation profiles may be simple, as indicated by the high accuracy achieved by the 1D approximated model of MLP (Table 4). It is necessary to perform a future study to evaluate the proposed method on other datasets with a much higher dimensionality of input with complex interactions. Partially, the interpretable latent distribution may be brought by our formulation of the task, by assigning binary variables to the input, rather than the introduction of EIS. Then, the weight of the first matrix in the fully-connected layer can be taken as a set of feature embeddings. Second, we have not provided a theoretical background to support the rationale for the stable training process achieved by the addition of EIS. However, it may be a straightforward solution to improve the learning performance of Diet Networks, which can be restricted by a limited variability represented by the transposed data matrix, by introducing a small number of learnable parameters in the form of a diagonal matrix. Finally, we did not include any additional information on somatic mutations from the biomedical literature. We lacked an opportunity to map the feature importance factors extracted by Diet Networks with EIS onto known mutation profiles (such as driver mutation or passenger mutation in the development of lung cancer).

Despite these limitations, we believe that our findings are meaningful and worth reporting because this is the first study that compared the performance of Diet Networks and MLP with the same number of layers and nodes. Diet Networks with EIS is designed to be versatile and can easily be applied to tasks with other datasets. Further, it is essential to emphasize that the original implementation of Diet Networks could not perform well in terms of accuracy and stability even for the current task, despite the various learning configurations. Therefore, the modification by adding EIS to Diet Networks can be a simple but effective solution to improve the learning capacity of Diet Networks.

### 4.2. Conclusions

The introduction of EIS stabilized a training process of Diet Networks and achieved the same level of accuracy to MLP with the same number of layers and nodes. The model was applied to the task of classifying the histology of lung cancer, and it presented a list of gene symbols responsible for contributing to the prediction.

## Figures and Tables

**Table 1:** Number of learnable parameters of each network architecture.

Architecture	Number of Parameters
MLP (Baseline)	2,299,394
MLP + EIS (Baseline)	2,317,355
Diet Networks (Baseline)	227,970
Diet Networks + EIS (Baseline)	245,931

**Table 2:** Classification accuracy on the validation dataset. *, **, †, ‡ indicate the best configurations for each network architecture.

Configuration	Accuracy ± Standard Deviation
Diet Networks (Baseline)	0.78 ± 0.06
Diet Networks (γ = 0.1) *	0.78 ± 0.05
Diet Networks (γ = 0.01)	0.67 ± 0.16
Diet Networks (γ = 0.001)	0.73 ± 0.17
Diet Networks + EIS (Baseline)	0.77 ± 0.04
Diet Networks + EIS (γ = 0.1) **	0.79 ± 0.02
Diet Networks + EIS (γ = 0.01)	0.77 ± 0.03
Diet Networks + EIS (γ = 0.001)	0.78 ± 0.02
Diet Networks + EIS (δ = 0.01)	0.76 ± 0.06
Diet Networks + EIS (δ = 0.001)	0.78 ± 0.03
Diet Networks + EIS (δ = 0.0001)	0.76 ± 0.03
Diet Networks + EIS (δ = 0.001, γ = 0.1)	0.76 ± 0.03
Diet Networks + EIS (δ = 0.001, γ = 0.01)	0.75 ± 0.03
Diet Networks + EIS (δ = 0.001, γ = 0.001)	0.75 ± 0.04
MLP (Baseline) †	0.82 ± 0.04
MLP + EIS (Baseline) ‡	0.77 ± 0.03
MLP + EIS (δ = 0.01)	0.74 ± 0.02
MLP + EIS (δ = 0.001)	0.75 ± 0.03
MLP + EIS (δ = 0.0001)	0.76 ± 0.04

**Table 3:** Ten most frequent dominant somatic mutations for each histology.

(**a**) Lung squamous cell carcinoma (LUSC)-dominant.	
**Gene Symbol**	***p*** **-Value**
*TP53*	8.1 $\times 10^{-23}$
*SYNE1*	1.1 $\times 10^{-14}$
*TTN*	1.5 $\times 10^{-11}$
*PTEN*	1.7 $\times 10^{-9}$
*NFE2L2*	3.1 $\times 10^{-9}$
*KMT2D*	6.1 $\times 10^{-9}$
*CDKN2A*	8.9 $\times 10^{-7}$
*LRRK2*	4.1 $\times 10^{-5}$
*PHC3*	7.0 $\times 10^{-5}$
*ATP10A*	1.1 $\times 10^{-4}$
(**b**) Lung adenocarcinoma (LUAD)-dominant.	
**Gene Symbol**	***p*** **-Value**
*KRAS*	6.0 $\times 10^{-32}$
*STK11*	3.6 $\times 10^{-12}$
*EGFR*	2.6 $\times 10^{-10}$
*PTPRD*	1.5 $\times 10^{-6}$
*SNTG1*	9.0 $\times 10^{-6}$
*RP1L1*	1.1 $\times 10^{-5}$
*NID1*	2.5 $\times 10^{-5}$
*LPPR4*	3.0 $\times 10^{-5}$
*SETBP1*	3.7 $\times 10^{-5}$
*FRMPD4*	6.9 $\times 10^{-5}$

**Table 4:** Validation accuracy based on the various principal component analysis (PCA) approximations of each network architecture.

Approximation	K=(1)	K=(2)	K=(1,2)
MLP (Baseline)	0.81 ± 0.04	0.52 ± 0.04	0.81 ± 0.04
MLP + EIS (Baseline)	0.76 ± 0.01	0.46 ± 0.04	0.76 ± 0.02
Diet Networks (γ = 0.1)	0.66 ± 0.19	0.48 ± 0.05	0.67 ± 0.11
Diet Networks + EIS (γ = 0.1)	0.56 ± 0.12	0.66 ± 0.13	0.80 ± 0.02

**Table 5:** Ten most frequent somatic mutations having (**a**) positive or (**b**) negative principal component (PC) scores for the second principal component.

(**a**) Positive PC scores.	
**Gene Symbol**	**PC Score**
*SYNE1*	6.49
*TP53*	5.95
*CUBN*	5.12
*MUC5B*	4.06
*PCDH15*	4.03
*TTN*	3.69
*TPTE*	3.65
*NFE2L2*	3.55
*TMEM132D*	3.40
*LRRK2*	3.19
(**b**) Negative PC scores.	
**Gene Symbol**	**PC Score**
*EGFR*	−4.73
*KRAS*	−4.29
*NID1*	−4.17
*GUCY2F*	−3.84
*ROBO2*	−3.11
*STK11*	−3.07
*FRMPD4*	−2.94
*PTPRD*	−2.57
*SETD2*	−2.49
*SIPA1L2*	−2.31
